# Choice of hospital after out-of-hospital cardiac arrest - a decision with far-reaching consequences: a study in a large German city

**DOI:** 10.1186/cc11516

**Published:** 2012-09-12

**Authors:** Jan Wnent, Stephan Seewald, Matthias Heringlake, Hans Lemke, Kirk Brauer, Rolf Lefering, Matthias Fischer, Tanja Jantzen, Berthold Bein, Martin Messelken, Jan-Thorsten Gräsner

**Affiliations:** 1 Department of Anesthesiology and Intensive Care Medicine, University Hospital Schleswig-Holstein, Campus Lübeck, Ratzeburger Allee 160 - Haus 13, 23538 Lübeck, Germany; 2 City of Dortmund, Fire Department, Steinstraße 25, 44122 Dortmund, Germany; 3 Institute for Research in Operative Medicine, University Witten/Herdecke - Faculty of Medicine, Ostmerheimer Straße 200 - Haus 38, 51109 Cologne, Germany; 4 Department of Anesthesiology and Intensive Care, Klinik am Eichert, Eichertstraße 3, 73035 Göppingen, Germany; 5 Intensive Care Transport Service Mecklenburg-Western Pomerania, German Red Cross (DRK) Parchim, Moltkeplatz 3, 19370 Parchim, Germany; 6 Department of Anesthesiology and Intensive Care Medicine, University Hospital Schleswig-Holstein, Campus Kiel, Schwanenweg 21, 24105 Kiel, Germany

## Abstract

**Introduction:**

Between 1 and 31% of patients suffering out-of-hospital cardiac arrest (OHCA) survive to discharge from hospital. International studies have shown that the level of care provided by the admitting hospital determines survival for patients suffering from OHCA. These data may only be partially transferable to the German medical system where responders are in-field emergency medical physicians. The present study determines the influence of the emergency physician's choice of admitting hospital on patient outcome after OHCA in a large urban setting.

**Methods:**

All data for patients collected in the German Resuscitation Registry for the city of Dortmund during 2007 and 2008 were analyzed. Patients under 18 years of age, with traumatic mechanism, and with incomplete charts were excluded. Admitting hospitals were divided into two groups: those without the capability for percutaneous coronary intervention (PCI), and those with PCI capability. Data were analyzed by multivariate statistics, taking into account the effects of mild therapeutic hypothermia treatment and PCI capability of the admitting hospital with respect to the neurological status upon hospital discharge.

**Results:**

Between 2007 and 2008 a total of 1,109 cardiopulmonary resuscitation attempts were registered for the city of Dortmund, of which 889 could be included in our study. Return of spontaneous circulation was achieved in 360 of 889 patients (40.5%). In total, 282 of 889 patients displayed return of spontaneous circulation during transport to the hospital (31.7%); 152 were transported with ongoing cardiopulmonary resuscitation (17.1%). Of the total 434 patients admitted to hospital, 264 were admitted to hospitals without PCI capability and 170 to hospitals with PCI capability. Multivariate analysis demonstrated a significant influence on patient discharge with good neurological status for those admitted to PCI hospitals (odds ratio 3.14 (95% confidence interval 1.51 to 6.56)), independent of receiving mild therapeutic hypothermia and/or PCI. Compared with patients admitted to hospitals without PCI capability, significantly more patients in PCI hospitals were discharged alive (41% vs. 13%, *P *< 0.001) and remained alive 1 year after the event (28% vs. 6%, *P *< 0.001).

**Conclusions:**

The choice of admitting hospital for patients suffering OHCA significantly influences treatment and outcome. This influence is independent of PCI performance and of mild therapeutic hypothermia. Further analysis is required to determine the possible parameters determining patient outcome.

## Introduction

The process of resuscitation after sudden cardiac arrest is an integral component of pre-hospital in-field emergency medical management and training, and, over the past decades, has been the subject of increasing research efforts in emergency medicine [1,2]. New scientific understanding is continually reflected in updated cardiopulmonary resuscitation (CPR) guidelines from the International Liaison Committee on Resuscitation. In spite of improved in-field emergency medical management, a noticeable improvement in the percentage of patients discharged from hospital with good neurological function has not been achieved over past decades [1]. Indeed, the percentage of patients discharged alive from hospital after out-of-hospital cardiac arrest (OHCA) varies between 1 and 31%, depending on study design and geographical region of study [1-4]. Reflecting these data, in-hospital clinical management of patients after OHCA plays an increasingly important role [5-7].

Several studies have demonstrated that outcome after OHCA can be improved with the implementation of mild therapeutic hypothermia [8-10] as well as percutaneous coronary intervention (PCI) [11-13]. In addition, both procedures may be combined [11,14,15]. In a study by Stub and colleagues, a greater proportion of patients were discharged home after OHCA when admitted to a facility with PCI capability [16]. This study, however, did not consider the performance of either PCI or mild therapeutic hypothermia. In addition, further studies were unable to conclusively corroborate this finding [17-19]. In general, studies to date do not sufficiently consider the in-hospital management of these patients; moreover, they cannot be extrapolated to the emergency medical system implemented in Germany, which provides for an on-site emergency physician. Furthermore, long-term survival data from patients after OHCA are lacking.

This study examines the influence of admitting hospital on patient survival after OHCA in the setting of a large German city. We estimate no difference in patient outcome between admission to PCI hospitals and non-PCI hospitals, except for patients receiving PCI or mild therapeutic hypothermia.

## Materials and methods

### Ethical approval

This study was approved by the ethical committee of the University of Lübeck (Record Number 11-050) and the scientific committee of the German Resuscitation Registry in compliance with current publication guidelines. Since cardiac arrest patients or their representative will mostly not be able to provide informed consent prior to treatment, the German Resuscitation Registry is generally conducted under federal regulations that allow a waiver of informed consent comparable with the Resuscitation Outcome Consortium funded by the National Institutes of Health. The Food and Drug Administration in 1996 developed specific regulations to permit research without prospective consent under carefully controlled circumstances. Secondly, any prerequisite condition of written informed consent for participation in the registry may lead to important additional selection bias. In addition, patient informed consent was waived by the ethics committee of the University of Cologne, Faculty of Medicine (Record Number 11-014).

### Emergency medical service system in Germany and Dortmund

The city of Dortmund (280.25 km²) is situated in the Ruhr valley, the most densely populated region of Europe. In 2008 the city registered over 580,500 inhabitants (48.5% male), an average population density of 2,080 inhabitants/km^2^.

In Germany, the in-field emergency medical service (EMS) is provided by a two-tier system: one unit comprises physician staffing, and the second only nonphysician staff. Both units are simultaneously dispatched for a CPR call, and are to arrive on scene at the same time. The physician as well as nonphysician staff are especially trained in emergency medicine procedures, including CPR, and provide advanced cardiac life support in accordance with current practice guidelines, including defibrillation.

The laws regulating the EMS in North Rhein-Westphalia stipulate a response time (call to response) not to exceed 8 minutes in at least 90% of dispatches. To fulfill this stipulation, the city of Dortmund maintains 17 emergency transport vehicles (25,653 unit hours/100,000 inhabitants and year) and five emergency physician vehicles (7,545 unit hours/100,000 inhabitants and year). In addition, a first-responder system is also in effect, with the aid of volunteer and professional fire department personnel.

### Hospital system in Dortmund

The EMS of Dortmund admits patients after OHCA to hospitals providing all levels of care. Seven of 22 hospitals also provide PCI capability.

### Data management

Different registries exist worldwide for the management of resuscitation data [20-23]. In 2007 the German Society of Anesthesiology and Critical Care (Deutsche Gesellschaft für Anästhesiologie und Intensivmedizin) instituted the national German Resuscitation Registry to manage (anonymously) data from patients suffering sudden cardiac arrest [24,25].

Data are collected at three different time points in accordance with the Utstein style protocol [26-28]. Initial treatment comprises initial resuscitation management administered by the emergency physician and emergency medical personnel as well as initial outcome, and finishes with hospital admission [29]. These documented field data were retrospectively supplemented with data from the emergency physician chart documentation. Secondly, post-resuscitation care comprises the admission status to hospital as well as in-hospital diagnostic procedures and treatment protocols. In addition, the discharge date along with neurological outcome are documented [30]. The latter is accomplished with the aid of five Cerebral Performance Categories (CPC) [31]. These data were collected retrospectively from the in-patient hospital charts. Finally, long-term care of survival comprises survival and assessment of quality of life 1 year after discharge by the patient's general practitioner [30].

### Statistical approach

We included all patients resuscitated by the EMS of Dortmund between the years 2007 and 2008. Excluded were patients under 18 years of age, and those with cardiac arrest secondary to traumatic injury. These patients are the responsibility of a supraregional care center. We also excluded patients whose charts were incomplete with respect to location of arrest, initial ECG, cause of cardiac arrest or initial post-resuscitation outcome.

Prospective regression analysis was used to determine the influence of choice of admitting hospital on the variables discharge alive and discharge with good neurological outcome (CPC 1 and CPC 2). Following univariate analysis to determine the influence of the factors age, sex, scene, and initial ECG rhythm on resuscitation outcome, multivariate analysis was performed on all variables with significant influence and established in-hospital treatments (PCI and hypothermia) after dividing the admitting hospitals into those without PCI capability (15 hospitals) and those with PCI capability (seven hospitals).

In addition, both univariate and multivariate analyses were performed to determine factors (patient demographics, EMS management) influencing choice of admitting hospital. Moreover, a univariate analysis was performed on long-term outcome data as well as in-hospital treatment capabilities for each admitting hospital group.

For binary and categorical variables, the chi-squared test and Fisher's exact test were used. For continuous variables (age and time), the Mann-Whitney U-test was used. Statistical analysis was performed with SPSS version 18 (SPSS Inc., Chicago, IL, USA). The level of significance was *P *< 0.05 with the confidence interval (CI) at 95%.

## Results

During the study period, 1,109 patients underwent CPR treatment by the EMS following OHCA. A total of 220 cases (19.8%) were excluded from the study. Out of these 220, 14 patients were under the age of 18 and 36 patients suffered from cardiac arrest secondary to traumatic injury. The remaining 170 patients were excluded due to incomplete data.

**Figure 1:**
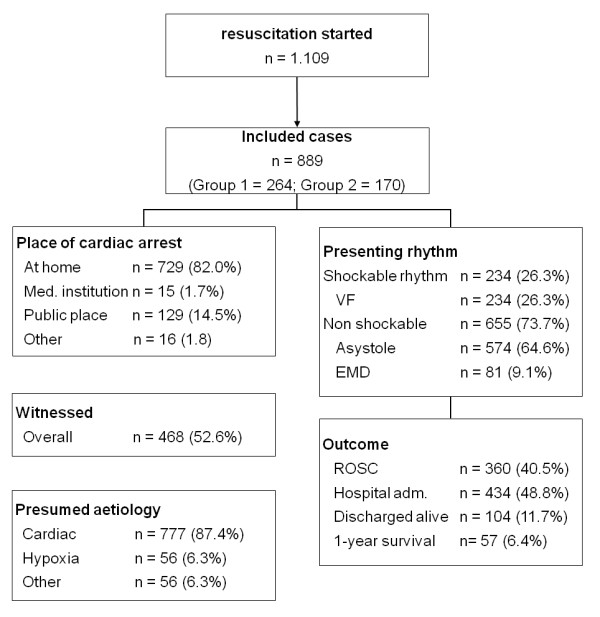
**Out-of-hospital cardiac arrest in the city of Dortmund between the years 2007 and 2008**. EMD, electromechanical dissociation; ROSC, return of spontaneous circulation; VF, ventricular fibrillation.

**Figure 2:**
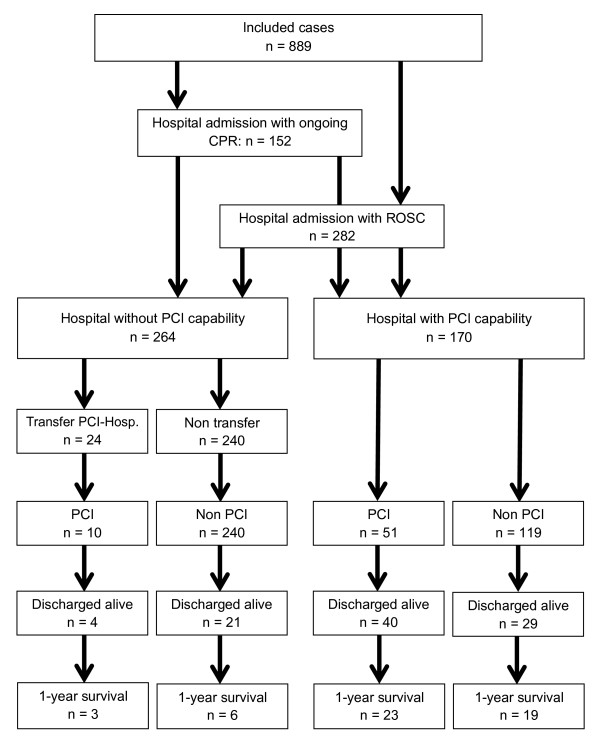
**Flow chart for patients after out-of-hospital cardiac arrest in the city of Dortmund**. CPR, cardiopulmonary resuscitation; OHCA, out-of-hospital cardiac arrest; PCI, percutaneous coronary intervention; ROSC, return of spontaneous circulation.

A total of 889 patient charts (80.2%) were included for analysis. The majority of patients were male (*n *= 562, 63.2%), and the average age was 69.4 years (standard deviation 14.5). A shockable rhythm was present in 234 cases (26.3%). Circulatory arrest was witnessed in a total of 468 cases (52.6%); bystander CPR was attempted in 117 cases (13.2%). In 777 cases (87.4%) a cardiac cause of arrest was presumed by the EMS team. Return of spontaneous circulation was achieved in a total of 360 cases (40.5%). Of total admissions to hospital, 282 patients were admitted with return of spontaneous circulation while 152 were admitted with CPR in progress. Out of all patients admitted, 104 were later discharged alive (Figures 1 and 2).

### Admitting hospital distribution criteria

In total, 264 out of 434 patients (60.8%) were admitted to a hospital without PCI capability and 170 patients (39.2%) were admitted to a hospital with PCI capability. The PCI hospital group had a higher proportion of male patients (71.6% vs. 61.3%, *P *= 0.03) and younger patients (64.7 years vs. 69.4 years, *P *= 0.002). Patients in PCI hospitals were more frequently considered to have a cardiac cause (92.9% vs. 83.0%, *P *= 0.01). For patients admitted during resuscitation in progress, more often a hospital without PCI capability was chosen (40.9% vs. 27.4%, *P *= 0.005). Patients whose neurological assessment was poor (CPC 3) prior to arrest were also more likely to be admitted to a non-PCI hospital (15.1% vs. 3.1%, *P *< 0.001). The location of arrest, initial ECG rhythm, witness category as well as time *en route *displayed no significant difference with respect to admitting hospital choice (Table S1 in Additional File 1).

Binary regression analysis demonstrated an independent influence on choice of admitting hospital for the following factors: male sex, noncardiac cause of OHCA excluding trauma, transport with CPR in progress, as well as severe neurological compromise (CPC 3) prior to collapse (Table 1).

**Table 1:** Result of binary regression analysis on patient characteristics influencing choice of admitting hospital

	Hospital with PCI capability	
	
	Odds ratio (95%CI)	*P *value
Gender - male	2.06 (1.21 to 3.53)	0.008
Presumed etiology - not cardiac	0.37 (0.16 to 0.85)	0.020
Transport with ongoing CPR	0.33 (0.20 to 0.55)	<0.001
Neurological status prior to collapse - CPC 3	0.17 (0.06 to 0.51)	0.002
Not shown in equation	Age	

CI, confidence interval; CPC, cerebral performance categories; CPR, cardiopulmonary resuscitation; PCI, percutaneous coronary intervention.

### In-hospital differences in therapy and outcome

Table 2 reflects therapeutic interventions and outcome of patients admitted to the two different hospital groups. Of the 264 patients primarily admitted to a hospital without PCI capability, 24 (9.1%) were transferred. Of these transferred patients, 10 (42%) received PCI within 24 hours at the new facility, 14 (58%) were discharged alive and nine (43%) survived 1 year after discharge. Of the remaining patients in non-PCI hospitals, 21 (9%) were discharged alive and six (3%) survived 1 year after discharge (Table S2 in Additional File 1).

**Table 2:** In-hospital therapy and outcome after out-of-hospital cardiac arrest

	Hospital without PCI capability	Hospital with PCI capability	*P *value	Odds ratio (95% CI)
*n*	264	170		
Transferred patients	24 (9.1%)	9 (5.3%)	0.194	0.56 (0.25 to 1.23)
TEE/TTE <120 minutes	54 (20.5%)	80 (47.1%)	<0.001	3.46 (2.26 to 5.28)
Pacemaker <24 hours	4 (1.5%)	1 (0.6%)	0.653	0.39 (0.04 to 3.47)
Fibrinolysis <24 hours	15 (5.7%)	4 (2.4%)	0.148	0.40 (0.13 to 1.23)
PCI <24 hours	10 (3.8%)	51 (30.0%)	<0.001	10.89 (5.34 to 22.19)
MTH <24 hours	15 (5.7%)	18 (10.6%)	0.066	1.97 (0.96 to 4.02)
24-hour survival	93 (42.9%)	103 (66.0%)	<0.001	2.59 (1.69 to 3.97)
Time in ICU (days)	4.3 (7.1)	7.5 (10.9)	0.003	
If discharged alive	12.7 (11.0)	16.8 (14.7)	0.337	
Mechanical ventilation time (hours)	49.5 (130.2)	93.2 (144.0)	<0.001	
If discharged alive	169.9 (251.6)	168.1 (173.8)	0.387	
Complications	106 (40.2%)	96 (56.5%)	0.001	1.93 (1.31 to 2.86)
Implantation of ICD	9 (3.4%)	22 (12.9%)	0.001	4.21 (1.89 to 9.39)
Discharged alive	35 (13.3%)	69 (40.6%)	<0.001	4.47 (2.80 to 7.15)
Neurological outcome				
CPC 1 + 2	13 (40.6%)	38 (57.6%)	0.135	0.50 (0.21 to 1.19)
CPC 3 + 4	19 (59.4%)	28 (42.4%)		
1-year survival	15 (6.0%)	42 (28.4%)	<0.001	6.23 (3.31 to 11.73)

Data presented as *n *(%) or mean (standard deviation). CI, confidence interval; CPC, cerebral performance categories; ICD, implantable cardioverter-defibrillator; ICU, intensive care unit; MTH, mild therapeutic hypothermia; PCI, percutaneous coronary intervention; TEE, transesophageal echocardiogram; TTE, transthoracic echocardiogram.

Fifty-one (30%) patients admitted to a hospital with PCI capability actually received the procedure. Moreover, patients receiving PCI admitted to the PCI hospital group were significantly more frequently discharged alive (78.4% vs. 24.4%, *P *< 0.001) and more frequently survived 1 year thereafter (65.7% vs. 16.8%, *P *< 0.001) compared with those admitted to the PCI hospital group but not receiving PCI.

We further found that patients admitted to hospitals with PCI capability received active cooling more frequently than did those admitted to hospitals without PCI capability (10.6% vs. 5.7%, *P *= 0.07). The patient outcome in hospitals with PCI capability was significantly improved in comparison with those without, with respect to 24-hour survival (66.0% vs. 42.9%, *P *< 0.001), discharge alive (40.6% vs. 13.3%, *P *< 0.001) as well as 1-year survival (28.4% vs. 6.0%, *P *< 0.001) (Table 2).

### Factors influencing hospital discharge and neurological outcome

Binary logistic regression analysis confirmed the influence of the admitting hospital on the frequency of alive discharges. Furthermore, significance was noted relative to therapeutic PCI, mild therapeutic hypothermia, as well as the detection of asystole as the presenting ECG rhythm (Table 3; see also Table S3 in Additional File 1). The results for the binary logistic regression analysis of discharge from hospital with good neurological status (CPC 1/2; Table S4 in Additional File 1) are shown in Table 3.

**Table 3:** Influence of admitting hospital on alive discharges and discharges with good neurological status

	Discharged alive		Hospital discharge with good neurological status	
	
	Odds ratio (95% CI)	*P *value	Odds ratio (95% CI)	*P *value
Hospital with PCI capability	2.39 (1.33 to 4.28)	0.004	3.14 (1.51 to 6.56)	0.002
Coronary angiography	4.57 (2.20 to 9.50)	<0.001	6.16 (3.03 to 12.55)	<0.001
Therapeutic hypothermia	5.31 (1.91 to 14.77)	0.001	3.11 (1.26 to 7.69)	0.014
Presenting rhythm - asystole	0.46 (0.26 to 0.82)	0.008	-	-
Not shown in equation	Gender, neurological status prior collapse, age		Presenting rhythm, age, bystander CPR	

Results of binary regression analysis on the influence of admitting hospital on frequency of alive discharges and discharged with good neurological status. CI, confidence interval; CPR, cardiopulmonary resuscitation; PCI, percutaneous coronary intervention.

## Discussion

Achieving return of spontaneous circulation is only a first step for the patient after OHCA if a good neurological outcome shall be achieved. An intensive critical care therapy with the application of post-resuscitation bundles, especially mild therapeutic hypothermia and PCI, is required for the best possible outcome [6,7,32].

The European Resuscitation Council guidelines from 2005 advocate mild therapeutic hypothermia for all unresponsive patients after OHCA and ventricular fibrillation. Moreover, hypothermia is thought to also benefit those patients presenting with a nonshockable rhythm [33]. Application of mild therapeutic hypothermia has been considerably expanded in the European Resuscitation Council guidelines of 2010 [34]. Our data show that, independent of presenting ECG rhythm, mild therapeutic hypothermia exerted an influence on discharge with good neurological status (adjusted odds ratio 3.11 (95% CI 1.26 to 7.69), *P *= 0.01).

In more than 70% of OHCA, a cardiac etiology was probable [35]. The guidelines from 2005 advise considering PCI for patients with evidence of coronary artery occlusion [33]. This advice was further strengthened in the 2010 guidelines [34]. Our data show that, independent of the supposed etiology of OHCA, therapeutic PCI exerted a highly beneficial influence on discharge with good neurological status (adjusted odds ratio 6.16 (95% CI 3.03 to 12.55), *P *< 0.001).

A total of 170 patients were admitted to hospital after initial ventricular fibrillation in this study. Sixteen (9.4%) of these were treated with active cooling. However, independent of the initial presenting ECG rhythm, no significant increase in mild therapeutic hypothermia was detected for those patients admitted to a PCI hospital compared with those admitted to a non-PCI hospital (10.6% vs. 5.7%). This rate of mild therapeutic hypothermia is not in accordance with current guidelines, and furthermore is much lower than has been previously published in the German literature (roughly 25% in Germany) [15,36].

In this study, 61 patients (14.1%) received therapeutic PCI. A total of 264 patients (60.8%) were initially admitted to a hospital without PCI capability. Merely 9.1% of these were later transferred to a hospital with this diagnostic and therapeutic modality. Therefore, during the study period, the rate of PCI interventions was also lower than advised by international guidelines. Comparably, between 2004 and 2010 the rate for PCI intervention was roughly 22% in the German Resuscitation Registry data [15].

In addition to the benefit achieved during post-resuscitation care through administration of PCI and mild therapeutic hypothermia, the in-hospital availability of these procedures alone seems to benefit resuscitation outcome. In comparison with hospitals without PCI capability, more patients were discharged alive (40.6% vs. 13.3%, *P *< 0.001) and achieved 1-year survival (28.4% vs. 6.0%, *P *< 0.001) after discharge from hospitals with PCI capability. Indeed, this beneficial influence on discharge from hospitals with PCI capability is independent of the actual administration of PCI and mild therapeutic hypothermia with regard to alive discharge rate (adjusted odds ratio 2.39 (95% CI 1.33 to 4.28), *P *= 0.004). Moreover, the same result was obtained when comparing discharge with good neurological status (adjusted odds ratio 3.14 (95% CI 1.51 to 6.56), *P *= 0.002).

The reasons for these discrepancies in outcomes between hospital with or without PCI capabilities cannot be determine exactly by the nature of this observational study. However, the improved outcomes after OHCA in hospitals with PCI capability may be related to a different expertise in cardiology and critical care as well as better critical care staffing and infrastructure. This better expertise could probably explain the significantly higher proportion of patients receiving an echocardiogram (transesophageal or transthoracic) in PCI hospitals (47.1% vs. 20.5%, *P *< 0.001). Perhaps PCI hospitals offer more diagnostic and specific treatment to patients after OHCA. For example, the implantable cardioverter-defibrillator implantation rate is significant higher in the PCI hospital group compared with non-PCI hospitals (12.9% vs. 3.4%, *P *= 0.001).

The findings of the present study are in line with recent observations from Victoria, Australia. Stub and colleagues showed that admission to a hospital with 24-hour PCI availability is associated with a higher discharge rate [16]; however, no mention was made of the performance of this procedure and the subsequent influence on outcome.

A univariate analysis on 4,087 patients included in the ROC Arrest Epidemiological Registry (Epistry) in the USA and Canada demonstrated a greater survival for patients admitted to hospitals with PCI capability [19]. However, multivariate analysis of the same data was unable to confirm these results. The conclusions from this analysis are furthermore constrained due to the large geographical heterogeneity and the lack of in-hospital treatment data.

Other studies confirm the strong influence of post-resuscitation care on survival, and interpret such a sequence as a chain of survival [5,7,17,32,37]. Our data also demonstrate that, even without complete adherence to treatment guidelines, availability of PCI greatly influences outcome after OHCA. Furthermore, we could demonstrate that great effort is necessary to improve successful implementation of the latest guidelines in the clinical, emergency setting. In the same fashion as a specialized trauma network along with dedicated trauma centers and treatment guidelines was created, so should a dedicated network be created for the specialized treatment of patients after OHCA [38,39]. In addition, the EMS providers should be more aware and involved in the development of local and regional admissions criteria in view of their responsibility in choosing the admitting hospital.

During the time of this study, a difference in patient demographics admitted to the different hospitals with and without PCI capability could be noted. Indeed, patients generally admitted to hospitals with PCI capability were younger (64.7 years vs. 69.4 years, *P *= 0.002) and more often male (71.6% vs. 61.3%, *P *= 0.03). Furthermore, patients with cardiac etiology for OHCA (92.9% vs. 83.0%, *P *= 0.01) and good neurological status prior to the event (CPC 1/2; 96.9% vs. 84.9%, *P *< 0.001) were also more often admitted to hospitals with PCI capability. The PCI hospital group therefore more often received patients with better prognosis.

In view of these findings, it would perhaps be important to consider, on a case by case basis, whether patients with a possibly worse prognosis should also be offered the best possible intensive care therapy provided at hospitals with PCI capability. Proper selection criteria ought be sought, to make such provision feasible in the future.

With the application of such selection criteria and hence admission of 434 patients to a hospital with PCI capability, it may have been possible to discharge up to 72 more patients alive (that is, 176 instead of 104 patients), and to achieve a 1-year survival for 66 patients (that is, 123 instead of 57 patients) .

### Limitations

Owing to the geographical constraints of this study (large German city), several limitations are apparent. The results of this study may be extrapolated only reservedly to other regions with differing healthcare infrastructure. In rural settings, for example, transport time to a hospital with PCI capability will be an important consideration when choosing the admitting hospital. This consideration could not be studied with our current data. Furthermore, in our study, only specific pre-hospital and in-hospital therapies were selectively highlighted. Indeed, there exist further important key issues in clinical management after OHCA, such as blood glucose level control, body temperature control, as well as seizure control [34], all of which are not considered presently. But this problem is related to most of the published studies. Every registry study has to deal with the discrepancy between detailed study documentation and practicality for the participants.

Future studies with a greater study population are therefore necessary to determine, in more detail, the influence exerted by in-hospital treatment on patients after OHCA.

## Conclusions

Patients being treated in hospitals with PCI capability have a better outcome compared with those treated in non-PCI hospitals. This finding is independent of PCI performance. By choosing the admitting hospital, the EMS provider directly influences therapeutic options as well as patient survival. Further, it is apparent that improved implementation of guidelines, especially relating to the application of PCI and mild therapeutic hypothermia, should be sought.

## Key messages

• Both PCI and mild therapeutic hypothermia are not implemented frequently enough and are not in accordance with the guidelines.

• The procedure of PCI and mild therapeutic hypothermia are independent predictors of alive discharge and discharge with good neurological status.

• Admission to a hospital with PCI capability is an independent predictor of discharge with good neurological status, regardless of implementation of either PCI or mild therapeutic hypothermia.

• The EMS providers substantially influence patient survival directly with their choice of admitting hospital.

## Abbreviations

CI: confidence interval; CPC: cerebral performance categories; CPR: cardiopulmonary resuscitation; EMS: emergency medical service; OHCA: out-of-hospital cardiac arrest; PCI: percutaneous coronary intervention.

## Competing interests

The authors declare that they have no competing interests.

## Authors' contributions

JW and SS made substantial contributions to conception and design, and drafted the manuscript. RL provided statistical support. J-TG conceived of the study, participated in its design and coordination, and helped to draft the manuscript. MH, HL, KB, MF, TJ, BB and MM were involved in the internal reviewing process. All authors read and approved the manuscript for publication.

## Supplementary Material

Additional file 1 **Table S1 presenting admitting hospital distribution criteria**. Table S2 presenting in-hospital therapy and outcome of patient primarily admitted to a hospital without PCI capability. Table S3 presenting factors influencing hospital discharge after OHCA. Table S4 presenting factors influencing neurological outcome after OHCA. Click here for file
