# Supplementary material for: Choice of hospital after out-of-hospital cardiac arrest - a decision with far-reaching consequences: a study in a large German city
Source: Crit Care. 2012 Sep 12;16(5):R164. doi: 10.1186/cc11516 (PMC3682259; doi:10.1186/cc11516)
Supplement: Additional file 1 — Table S1 presenting admitting hospital distribution criteria. Table S2 presenting in-hospital therapy and outcome of patient primarily admitted to a hospital without PCI capability. Table S3 presenting factors influencing hospital discharge after OHCA. Table S4 presenting factors influencing neurological outcome after OHCA. [file cc11516-S1.PDF]

Table S1: Admitting hospital distribution criteria.

	hospital without PCI-capability (Group 1)	hospital with PCI-capability (Group 2)	P value	OR (95%CI)	Test method
**n**	264	170			
**Gender male**	160 (61.3%)	121 (71.6%)	0.030	0.63 (0.41-0.95)	Fisher exact
**Age** (years): Mean (SD)	69.4 (13.6)	64.7 (15.1)	0.002		U-Test
**Age**					
- 18 – 20 years	0	3 (1.8%)			
- 20 – 40 years	9 (3.4%)	12 (7.1%)			
- 40 – 60 years	60 (22.7%)	48 (28.2%)	0.006		Chi²
- 60 – 80 years	140 (53.0%)	88 (51.8%)			
- > 80 years	55 (20.8%)	19 (11.2%)			
**Location**					
- At home/Nursing home	199 (75.4%)	124 (72.9%)			
- Doctor's office / Medical institution	5 (1.9%)	7 (4.1%)	0.355		Chi²
- Public place	54 (20.5%)	32 (18.8%)			
- Others	6 (2.3%)	7 (4.1%)			
**Presenting rhythm**					
- Ventricular Fibrillation	92 (34.8%)	78 (45.9%)			
- EMD	31 (11.7%)	18 (10.6%)	0.068		Chi²
- Asystole	141 (53.4%)	74 (43.5%)			
**Witnessed**					
- None	86 (32.6%)	61 (35.9%)			
- Lay people	128 (48.5%)	80 (47.1%)	0.873		Chi²
- Professionals	50 (18.9%)	29 (17.1%)			
**Bystander CPR**	32 (12.2%)	23 (13.5%)	0.661	1.13 (0.64-2.01)	Fisher exact
**Presumed aetiology**					
- Cardiac	219 (83.0%)	158 (92.9%)			
- Hypoxia	26 (9.8%)	6 (3.5%)	0.010		Chi²
- Other not cardial	19 (7.2%)	6 (3.5%)			
**Qualification of emergency physician**					
- Junior doctor	101 (50.2%)	69 (52.3%)	0.738	0.92 (0.59-1.43)	Fisher exact
- Specialist	100 (49.8%)	63 (47.7%)			
**Field of emergency physician**					
- Internal medicine	54 (27.0%)	40 (30.8%)			
- Surgery	47 (23.5%)	24 (18.5%)	0.704		Chi²
- Anaesthesia	97 (48.5%)	65 (50.0%)			
- Paediatrics	2 (1.0%)	1 (0.8%)			
**Arrest to EMS arrival time** (min): Mean (SD)	7.1 (4.8)	7.4 (5.5)	0.962		U-Test

**Transport time** (min): Mean (SD)					
- with ongoing CPR	7.1 (7.2)	8.3 (7.0)	0.077		U-Test
- without CPR	10.6 (10.5)	11.0 (9.1)	0.417		U-Test
**ROSC**	201 (76.7%)	141 (82.9%)	0.145	1.48 (0.90-2.41)	Fisher exact
**Transport with ongoing CPR**	106 (40.9%)	46 (27.4%)	0.005	0.54 (0.36-0.83)	Fisher exact
**Neurological status prior collapse**					
- CPC 1	122 (70.9%)	93 (71.5%)			
- CPC 2	24 (14.0%)	33 (25.4%)	p<0.001		Chi²
- CPC 3	26 (15.1%)	4 (3.1%)			

(CPC, cerebral performance categories; CPR, cardiopulmonary resuscitation; EMD, electromechanical dissociation; EMS, emergency medical services; PCI, percutaneous coronary intervention; SD, standard deviation)

Table S2: In-Hospital therapy and outcome of patient primarily admitted to a hospital without PCI capability.

	**non transfer**	**transfer to PCI-hospital**
**n**	240	24
**TEE/TTE < 120 min**	39 (16.2%)	15 (62.5%)
**Pacemaker < 24h**	3 (1.2%)	1 (4.2%)
**Fibrinolysis < 24h**	13 (5.4%)	2 (8.3%)
**PCI < 24h**	0	10 (41.7%)
**MTH < 24h**	7 (2.9%)	8 (33.3%)
**24h survival**	74 (38.3%)	19 (79.2%)
**Complications**	93 (38.8%)	13 (54.2%)
**Implantation of ICD**	5 (2.1%)	4 (16.7%)
**Discharged alive**	21 (8.8%)	14 (58.3%)
**Neurological outcome**		
- CPC 1+2	6 (31.6%)	7 (53.8%)
- CPC 3+4	13 (68.4%)	6 (46.2%)
**1-year survival**	6 (2.6%)	9 (42.9%)

(CPC, cerebral performance categories; ICD, Implantable cardioverter-defibrillator; MTH, mild therapeutic hypothermia; PCI, percutaneous coronary intervention; TEE, transesophageal echocardiogram; TTE, transthoracic echocardiogram)

Table S3: Factors influencing hospital discharge after OHCA.

	Discharged alive	dead in hospital	P value	OR (95%CI)	Test method
**n**	104	330			
**Gender male**	78 (75.0%)	203 (62.3%)	0.018	1.81 (1.11-2.99)	Fisher exact
**Age** (years): means (SD)	62.5 (14.0)	69.2 (14.1)	p<0.001		U-Test
**Age**					
- 18 – 20 years	2 (1.9%)	1 (0.3%)			
- 20 – 40 years	3 (2.9%)	18 (5.5%)			
- 40 – 60 years	42 (40.4%)	66 (20.0%)	p<0.001		Chi²
- 60 – 80 years	49 (47.1%)	179 (54.2%)			
- > 80 years	8 (7.7%)	66 (20.0%)			
**Location**					
- At home/Nursing home	73 (70.2%)	250 (75.8%)			
- Doctor’s office/ Medical institution	4 (3.8%)	8 (2.4%)	0.470		Chi²
- Public place	22 (21.2%)	64 (19.4%)			
- Others	5 (4.8%)	8 (2.4%)			
**Presenting rhythm**					
- Ventricular Fibrillation	68 (65.4%)	102 (30.9%)			
- EMD	4 (3.8%)	45 (13.6%)	p<0.001		Chi²
- Asystole	32 (30.8%)	183 (55.5%)			
**Witnessed**					
- None	33 (31.7%)	114 (34.5%)			
- Lay people	54 (51.9%)	154 (46.7%)	0.638		Chi²
- Professionals	17 (16.3%)	62 (18.8%)			
**Bystander CPR**	18 (17.3%)	37 (11.2%)	0.127	0.60 (0.33-1.11)	Fisher exact
**Presumed aetiology**					
- Cardiac	92 (88.5%)	285 (86.4%)			
- Hypoxia	7 (6.7%)	25 (7.6%)	0.847		Chi²
- Other not cardial	5 (4.8%)	20 (6.1%)			
**Qualification of emergency physician**					
- Junior doctor	40 (47.6%)	130 (52.2%)	0.528	0.83 (0.51-1.37)	Fisher exact
- Specialist	44 (52.4%)	119 (47.8%)			
**Field of emergency physician**					
- Internal medicine	22 (26.2%)	72 (29.3%)			
- Surgery	17 (20.2%)	54 (22.0%)	0.379		Chi²
- Anaesthesia	43 (51.2%)	119 (48.4%)			
- Paediatrics	2 (2.4%)	1 (0.4%)			
**Arrest to EMS arrival time** (min): Mean (SD)	6.6 (3.1)	7.4 (5.5)	0.498		U-Test

**Neurological status prior collapse**					
- CPC 1	74 (76.3%)	141 (68.8%)			
- CPC 2	20 (20.6%)	37 (18.0%)	0.024		Chi²
- CPC 3	3 (3.1%)	27 (13.2%)			
**Hospital group**					
- Hospital without PCI-capability	35 (33.7%)	229 (69.4%)	p<0.001	0.22 (0.14-0.36)	Fisher exact
- hospital with PCI-capability	69 (66.3%)	101 (30.6%)			
**coronary angiography**	44 (42.3%)	17 (5.2%)	p<0.001	0.07 (0.04-0.14)	Fisher exact
**therapeutic hypothermia**	19 (18.3%)	14 (4.2%)	p<0.001	0.20 (0.1-0.41)	Fisher exact

(CPC, cerebral performance categories; CPR, cardiopulmonary resuscitation; EMD, electromechanical dissociation; EMS, emergency medical services; PCI, percutaneous coronary intervention; SD, standard deviation)

Table S4: Factors influencing neurological outcome after OHCA*.

* 6 patient charts were excluded because of incomplete data for the neurological outcome

	good neurological outcome (CPC 1+2)	bad neurological outcome or death (CPC 3-5)	P-values	OR (95%CI)	Test method
**n**	51	377			
**Gender male**	38 (74.5%)	238 (63.8%)	0.159	1.66 (0.85-3.22)	Fisher exact
**Age** (years): Mean (SD)	61.4 (14.9)	68.5 (14.1)	0.001		U-Test
**Age**					
- 18 – 20 years	2 (3.9%)	1 (0.3%)			
- 20 – 40 years	0	20 (5.3%)			
- 40 – 60 years	24 (47.1%)	82 (21.8%)	p<0.001		Chi²
- 60 – 80 years	21 (41.2%)	204 (54.1%)			
- > 80 years	4 (7.8%)	70 (18.6%)			
**Location**					
- At home/Nursing home	34 (66.7%)	286 (75.9%)			
- Doctor's office/ Medical institution	3 (5.9%)	8 (2.1%)	0.311		Chi²
- Public place	12 (23.5%)	72 (19.1%)			
- Others	2 (3.9%)	11 (2.9%)			
**Presenting rhythm**					
- Ventricular Fibrillation	33 (64.7%)	133 (35.3%)			
- EMD	2 (3.9%)	46 (12.2%)	p<0.001		Chi²
- Asystole	16 (31.4%)	198 (52.5%)			

**Witnessed**					
- None	16 (31.4%)	131 (34.7%)			
- Lay people	24 (47.1%)	180 (47.7%)	0.754		Chi²
- Professionals	11 (21.6%)	66 (17.5%)			
**Bystander CPR**	12 (23.5%)	43 (11.4%)	0.024	0.42 (0.20-0.86)	Fisher exact
**Presumed aetiology**					
- Cardial	44 (86.3%)	327 (86.7%)			
- Hypoxia	5 (9.8%)	27 (7.2%)	0.675		Chi²
- Other not cardial	2 (3.9%)	23 (6.1%)			
**Qualification of emergency physician**					
- Junior doctor	17 (44.7%)	149 (51.6%)	0.491	0.76 (0.39-1.50)	Fisher exact
- Specialist	21 (55.3%)	140 (48.4%)			
**Field of emergency physician**					
- Internal medicine	9 (23.7%)	84 (29.4%)			
- Surgery	10 (26.3%)	61 (21.3%)	0.792		Chi²
- Anaesthesia	19 (50.0%)	139 (48.6%)			
- Paediatrics	0	2 (0.7%)			
**Arrest to EMS arrival time** (min): Mean (SD)	6.3 (2.7)	7.4 (5.3)	0.274		U-Test
**Neurological status prior collapse**					
- CPC 1	40 (81.6%)	171 (68.7%)			
- CPC 2	9 (18.4%)	48 (19.3%)	0.032		Fisher exact
- CPC 3	0	30 (12.0%)			
**Hospital group**					
- Hospital without PCI-capability	13 (25.5%)	248 (65.8%)	p<0.001	0.18 (0.09-0.25)	Fisher exact
- hospital with PCI-capability	38 (74.5%)	129 (34.2%)			
**coronary angiography**	26 (51.0%)	33 (8.8%)	p<0.001	0.09 (0.05-0.18)	Fisher exact
**therapeutic hypothermia**	11 (21.6%)	22 (5.8%)	0.001	0.23 (0.10-0.50)	Fisher exact

(CPC, cerebral performance categories; CPR, cardiopulmonary resuscitation; EMD, electromechanical dissociation; EMS, emergency medical services; PCI, percutaneous coronary intervention; SD, standard deviation)
